# Investigation of Pore Size Effect on the Infiltration Process of Ti6Al4V/xAg Metal Matrix Composites

**DOI:** 10.3390/ma18050939

**Published:** 2025-02-21

**Authors:** Juan Israel Villa-Tapia, Héctor Javier Vergara-Hernández, Luis Olmos, Dante Arteaga, Jorge Sergio Téllez-Martínez, Víctor Manuel Solorio-García, Elena Mihalcea

**Affiliations:** 1 División de Estudios de Posgrado e Investigación, Tecnológico Nacional de México/I.T. Morelia, Av. Tecnológico #1500, Colonia Lomas de Santiaguito, Morelia C.P. 58120, Mexico; m14120336@morelia.tecnm.mx (J.I.V.-T.); hector.vh@morelia.tecnm.mx (H.J.V.-H.); jstellezm@gmail.com (J.S.T.-M.); v_ms_g@hotmail.com (V.M.S.-G.); 2 Instituto de Investigaciones en Ciencias de la Tierra (INICIT), Universidad Michoacana de San Nicolás de Hidalgo, Fco. J.Mujica S/N, Morelia C.P. 58060, Mexico; luis.olmos@umich.mx; 3 Centro de Geociencias, Universidad Nacional Autónoma de México, Blvd. Juriquilla No. 3001, Querétaro C.P. 76230, Mexico; darteaga@geociencias.unam.mx; 4 Unidad Académica de Ingeniería I, Universidad Autónoma de Zacatecas, Av. López Valverde 801, Zacatecas C.P. 98060, Mexico

**Keywords:** liquid phase sintering, pressureless infiltration, biomedical materials, permeability, computed microtomography, porous compacts

## Abstract

This work investigates the fabrication of Ti6Al4V composites manufactured by powder metallurgy through pressureless infiltration. Porous Ti6Al4V alloy compacts with different particle sizes were fabricated by sintering and then, liquid Ag was infiltrated to obtain composites. Computed microtomography was used to analyze the samples before and after infiltration. Numerical flow simulations and dilatometry tests evaluated the kinetics of Ag infiltration into porous Ti6Al4V compacts. Microstructure was observed by SEM and mechanical strength was evaluated by compression tests. Results showed that the pore properties play a crucial role in the infiltration timing and the distribution of the Ag’s liquid. In particular, large pores allowed the infiltration to start a few °C degrees earlier than samples with smaller pores. Three-dimensional images after infiltration showed that most of the pores were filled and the remaining ones were isolated. The resulting microstructure was composed of Ti_2_Ag, α-Ti and Ag phases, indicating that the Ag diffusion occurred. Furthermore, the mechanical strength depends on the interparticle neck sizes and the Ag improves the plastic deformation reached during compression tests. The best results were obtained for the samples with larger pore sizes because the resulting mechanical properties (E = 23 GPa and σ_y_ = 403 MPa) are close to that of human bones, making it the best candidate as an antibacterial material for biomedical use.

## 1. Introduction

Throughout the years, research on the use of metallic alloys in biomedical implants has been of great relevance, since these must comply with a certain series of characteristics and very specific parameters to be considered optimal for biomedical use. Different requirements are needed to use materials for long term orthopedic implants [1,2,3]. Among the most important are those related to the mechanical properties, which should have the most similar values possible to that of human bones. The main properties are low stiffness, high strength, and high hardness. In addition, those materials have to be biocompatible with the human body in order to avoid toxic components for the human body. In addition, those materials should demonstrate good corrosion behavior under biological fluids and, for materials used in bone joints, a wear resistance is required. In addition, it is highly recommended that materials pose a good antibacterial activity to avoid infections during the surgery process [4]. Finally, it is expected that materials also show a good osseointegration; this is reached by controlling the pore features to mimic the bone structure [5].

Currently, Ti alloys have had a great scientific development because they fulfill most of the medical requirements. These are widely used in different types of implants such as dental, external and craniofacial prostheses, and their use extends to surgical devices. The adaptation of Ti alloys for biomedical use is attributed to their high range of biocompatibility, which is the ability of a material to adapt to the human body without causing any adverse effects; this is in addition to generating an optimal cellular response so that the material in question is functional for the user. The Ti6Al4V alloy, composed of titanium (Ti), aluminum (Al) at 6% and vanadium (V) at 4%, meets the biocompatibility ranges necessary to be an applicable material for biomedical use based on the pH effects on it, as well as offering a unique combination of mechanical strength, light weight and excellent corrosion resistance [6,7].

Nevertheless, Ti6Al4V has the disadvantage of having very low antibacterial resistance, which directly affects its use as a biomedical material. It can lead to complications during and after the surgery due to bacterial infections which, directly compromises the useful life of the implant and the longevity of the human bone to which the implant is anchored [8]. In addition to this, most of the Ti6Al4V alloy parts for biomedical implants are manufactured with the sintering process due to the complex morphologies used, which results in parts with porosity and low density, also affecting their mechanical properties in the long term [9].

To overcome the issues associated with alloys, combinations with materials that have antibacterial activity, such as Ag, have been used [10,11,12,13]. It has been proven that TI6Al4V and Ag generate good antibacterial levels; M. Chen et al. [14] made the addition of Ag in different wt.% and found a higher bactericidal rate in the samples with higher wt.% of Ag added. This resulted in a considerable improvement in the alloys based on these elements for biomedical use. Different research with Ag addition [15] demonstrated a bactericidal rate around 99.99% for bacteria such as S. aureus and E. Coli, demonstrating that bacterial cell walls are completely altered by Ag.

Powder metallurgy is commonly used as a manufacturing process for biomedical prostheses because the powder sintering process has a lower production cost [16]. However, since it has a porous part, there is a risk of failure due to the mechanical behavior of these structures [17]. The addition of elements in metal matrix composites can be performed to reinforce them in different ways; an example is the assisted liquid infiltration which implies important improvements in the mechanical properties. Nevertheless, this technique has certain disadvantages such as the induction of microcracks which are detrimental to the composites [18]. On the other hand, with pressureless liquid infiltration, a satisfactory infiltration by capillary phenomena can be performed, obtaining good results without these structural defects since no external force is required to infiltrate the reinforcement material into the composite [19].

The principle of the infiltration technique is to pass some material through a porous medium in order to fill the pores [20]. Important aspects must be considered to have good infiltration levels such as wettability and permeability, in addition to inconsistencies derived from errors that may exist in the control of atmospheres and preparation of substrates that can lead to discrepancies in the contact angles. Sobczak et al. [21] concluded that it is necessary to have a control or knowledge of the surface and the atmosphere used to avoid those problems. Infiltration can be used as a method of densification of a porous material completely changing its properties. Kumar et al. [22] infiltrated a YBCO superconductor with Ag obtaining good levels of densification changing the pore volume fraction of the worked parts, in addition to their mechanical and microstructural properties. Arivazhagan et al. [23] made Mg infiltration without pressure into a porous Ti6Al4V compact, completely improving the maximum compressive strength in the infiltrated parts. The mechanical strength was improved, going from 178 MPa of the porous structures to 415 MPa of the infiltrated compacts, thus improving their mechanical properties and densification. For the infiltration into a porous compact to take place optimally, it depends on different variables—one of the most important being the existing permeability. Olmos et al. [24] carried out permeability studies in a Ti-6Al-4V porous compact through computerized microtomography, finding that the permeability is directly influenced by the pore volume fraction, the size of the channel, as well as the number and shape of the pores.

The Ti6Al4V/Ag combination, besides having good antibacterial properties, shows good results in terms of mechanical behavior. Solorio et al. [25] performed sintering of Ti6Al4V powders with different vol.% of Ag, in order to perform compression tests on the sintered compacts, obtaining better results with higher vol.% of Ag added, due to the fact that they presented densification values around 97%. The results of the combination of these materials are beneficial since their mechanical and antibacterial attributes are improved, allowing the development of a composite material with a specific microstructure which allows its use in biomedical implants [26]. The wettability of the Ag on the Ti or Ti alloys has not been studied from the wetting angle; nevertheless, it was reported that titanium is immiscible with different metals [26] as Ag [27]. In where a liquid Ag form during sintering enhanced the densification of composites. In both cases, the wettability of the alloy increased, which positively influenced the biological response of the implant.

As demonstrated, the Ti6Al4V/Ag combination is suitable for use in biomedical implants; however, it has a weakness in the density levels that it presents once sintered, which is a serious problem that compromises the life of a prosthesis. This can be solved with techniques such as gravity infiltration, thus being able to improve the mechanical properties without neglecting the antibacterial part. Since this option has not yet been studied, the selection of this technique allows it to be developed in its entirety. Therefore, the main objective of this work is to analyze the infiltration process from the point of view of the void space to be filled by liquid. Thus, porous compacts with different pore characteristics were prepared to evaluate the flow throughout the porosity with the aim of fabricating Ti6Al4V/Ag composites that could be used as bone implants. Additionally, a microstructural characterization and mechanical properties were evaluated in order to determine the viability for use them as orthopedic implants.

## 2. Materials and Methods

### 2.1. Raw Materials and Porous Compacts Fabrication

**Figure 1:**
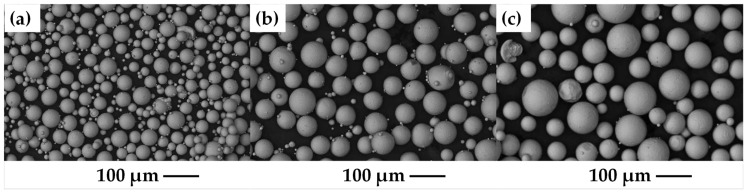
Initial powders Ti6Al4V, (**a**) 20–45 µm, (**b**) 45–75 µm and (**c**) 75–106 µm.

**Figure 2:**
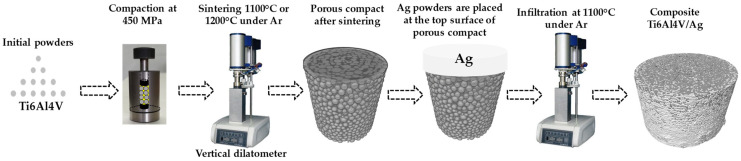
Schema of the experimental set up.

Atomized powders with spherical shapes of the Ti6Al4V alloy, produced by Raymor, Boisbriand, Canada, were sieved by passing them through meshes of different sizes, and different particle sizes were obtained: 20–45 μm, 45–75 μm and 75–106 μm. The powders obtained from the sieving process are shown in Figure 1a–c, respectively, these have different particle size percentages within the previously established ranges. Once the powders were separated, each particle size was mixed with PVA (polyvinyl alcohol) as a binder in 1% by weight solution, and then poured into a cylindrical stainless-steel die with a diameter of 6 mm and compacted at 450 MPa in an Instron 1195 mechanical testing machine to obtain green compacts. The green compacts were put through the sintering process, in which a different thermal cycle was given. Compacts with a particle size of 20–45 μm were sintered at a temperature of 1100 °C, while compacts of 45–75 μm and 75–106 μm were sintered at 1200 °C. A heating rate of 20 °C/min and the dwell time was 1 h for all samples. The sintering temperatures were chosen because a full interconnected porosity is desired to achieve a good infiltration of Ag liquid. Before sintering, PVA was removed at 500 °C for 45 min. The whole thermal cycle was carried out under atmosphere of high purity argon in a Linseis L75V vertical dilatometer, See Figure 2. To determine the density of each sintered compact, precise measurements of its mass and dimensions were performed.

### 2.2. Characterization of Porous Compacts

The sintered compacts were cut into pieces of 2 mm by 2 mm on a Struers Accutom-2 precision cutter (Struers S.A.S., Champigny sur Marne cedex, France) and then microtomography was performed in a Versa 510, Zeiss nanotomograph (Carl-Zeiss-Strasse, Oberkochen, Germany), in which separate sessions were performed for each of the compacts with different particle sizes, obtaining a file with all the projections for each compact. In the image analysis, the microtomography images were converted into binary images through filters, thresholding and segmentation processing [28]. This allowed us to obtain the formation of the interparticle necks generated in the compacts. The software calculated the surfaces of the lines that divide the particles in the image, to adjust the contrast of the image itself, thus reducing the number of errors that it could contain. The analysis of the resulting microstructure in each of the compacts was obtained from 3D images with a voxel size of 3.5 μm, being worked in Avizo 2019.1^®^ software, in addition to performing permeability simulations; these operate with binary images where the pores are represented with an intensity of 1 and Ti6Al4V with an intensity of 0. The simulation model worked in Avizo^®^ solves the Navier–Stokes equations for laminar flow and is governed by Darcy’s Law for absolute permeability. The boundary conditions that were set to the software are the inlet and outlet pressure being 130 and 100 kPa, respectively, and the viscosity of the fluid with a value of 0.00327 Pa.s, which in this case was Ag at 1100 °C, being the actual process temperature [29].

### 2.3. Infiltration Process and Composite Characterization

The infiltration of Ag in the sintered compacts was carried out again in the vertical dilatometer where the sintering was performed. Figure 2 illustrates the experimental steps followed to fabricate the composites by infiltration. The aim to perform the infiltration in the dilatometer was to measure the temperature at which the liquid is infiltrated into the porous compact by in situ following the axial displacement. The infiltration set up was based on having the porous Ti6Al4V compact inside a zirconium crucible of 10 mm diameter in the lower part and in the upper part of the compact the silver compacted in the form of a pellet, in addition to this an alumina rod of 1 mm in diameter and 2 cm high. All the compacts followed the same thermal cycle, being taken to a temperature of 1100 °C with a dwell time of 5 min and a heating rate of 25 °C/min. The compacts infiltrated with silver were again characterized by microtomography to perform image analysis and observe the silver infiltrated in each of the compacts. In addition, the microstructure of the infiltrated compacts was observed by scanning electron microscopy, where all the compacts were mounted on Bakelite for better handling due to the size of the pieces and prepared by roughing with SiC abrasive sandpaper starting at sandpaper 320 and ending at 2000, to be polished and reach the mirror surface using 1 µm alumina as an abrasive medium. The pieces were observed under a scanning electron microscope at 20 kV, in order to analyze the phases obtained. Compacts 6 mm wide and 5 mm high, sintered and infiltrated with silver under the same conditions as the cut compacts were subjected to mechanical compression tests in order to perform a strength evaluation. The test consisted of placing the compacts on the base of the testing machine previously used for compacting powders and then applying compression pressure until the fracture point was reached.

## 3. Results and Discussion

### 3.1. Sintering

**Figure 3:**
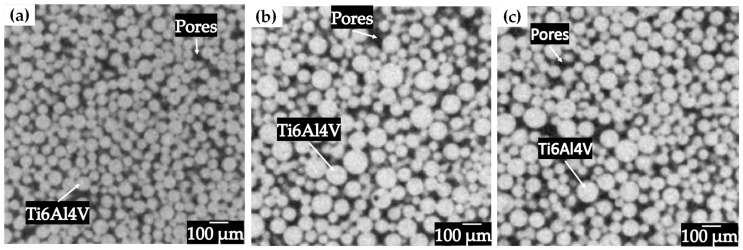
2D virtual slices of Ti6Al4V sintered (**a**) 20–45 µm 1100 °C, (**b**) 45–75 µm 1200 °C and (**c**) 75–106 µm 1200 °C.

Compacts with different particle sizes were sintered at different temperatures in order to obtain different levels of densification, as it was reported for solid state sintering of Ti6AlV powders with different particle sizes [30]. The porous structures obtained in the sintering process are shown for each particle size in Figure 3a–c, observing a structure consisting of Ti6Al4V particles and pores. It can be seen that the size of the powders used influences the volume fraction of pores obtained, which was expected due to the sintering temperatures used. For the compact fabricated with the particle size distribution of 20–45 µm, the lowest volume fraction was obtained because the contact area at the neck level is greater, which results in a higher densification, reaching a relative density of 82.5%. Samples with a particle size distribution of 45–75 µm reached a relative density of 78.7% meanwhile the relative density of samples fabricated with powders of 75–106 m reached a relative density of 70.3%. The relationship between the particle size and the densification is direct since it is observed that having a larger particle size results in a lower densification and; therefore, a higher volume fraction of pores is obtained, due to the fact that it has a smaller contact area at the interparticle necks [31].

**Figure 4:**
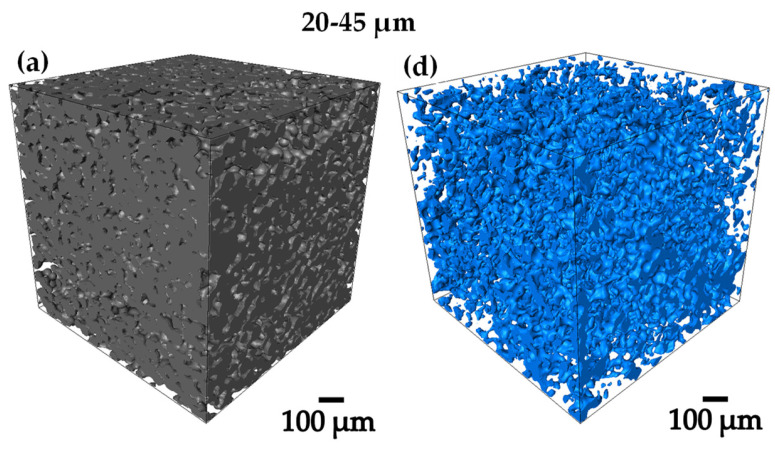

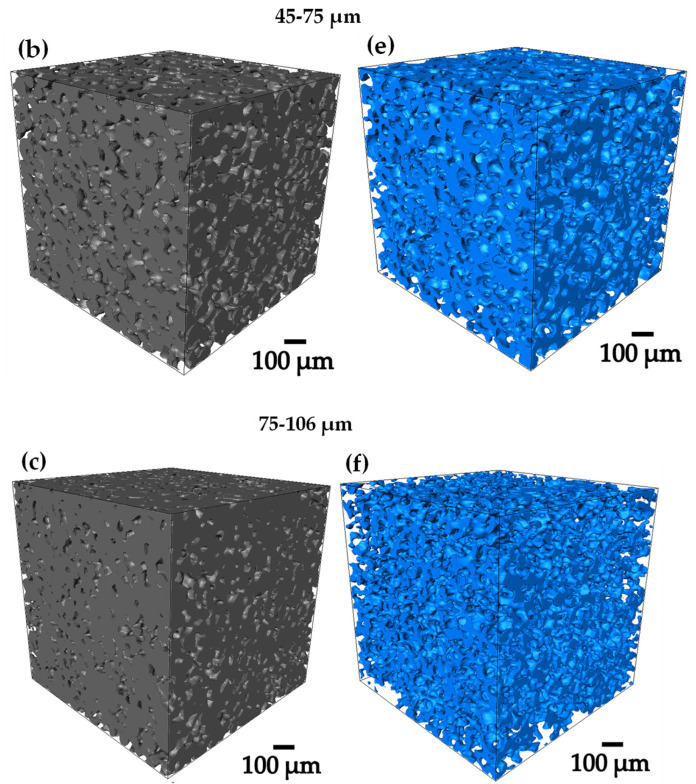
3D rendering of the sintered compacts showing 2 different phases (**a**–**c**) show the Ti6Al4V distribution and (**d**–**f**) show the pore distribution obtained.

From 3D images of gray level acquired by computed microtomography, binary images were obtained by thresholding, in order to be able to work on the whole 3D image analysis [32,33]. Figure 4a–f, shows the 3D renderings of the pre-sintered compacts, in where the Ti6Al4V and the porosity can be qualitatively observed [34]. The 75–106 µm compact was the one that presented the highest volume fraction of pores with 29.6% as shown in Table 1, denoting the previously mentioned. All the compacts presented a pore volume fraction between 15 and 30 Figure 4a–f, obtaining different levels of densification to perform the infiltration of the Ag.

**Figure 5:**
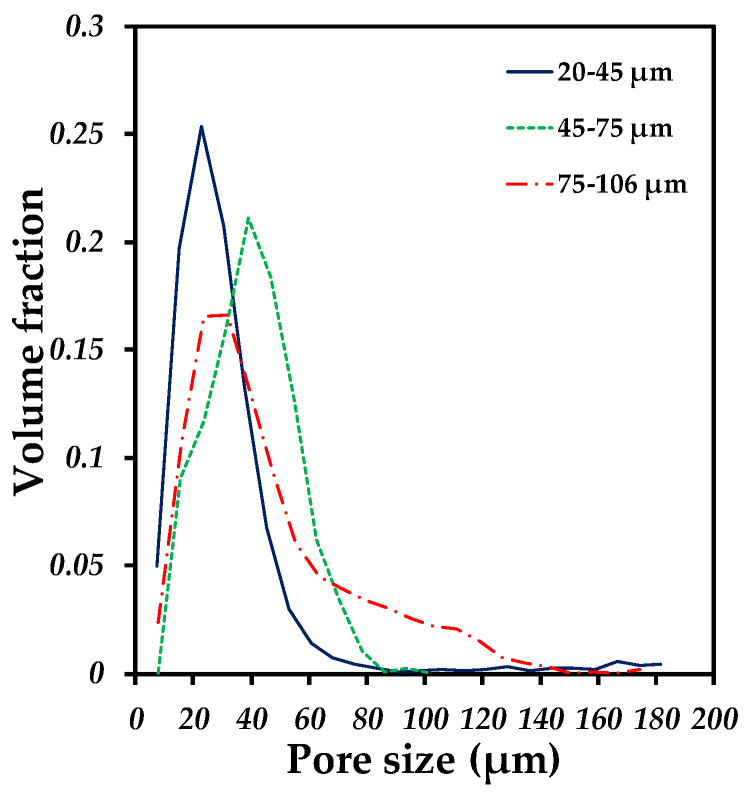
Pore size distribution of porous compacts fabricated with different particle size of Ti6AlV powders.

The segmented images were used to obtain the pore size distribution and the image analysis was performed in which the data obtained in the images were converted into tangible numerical data and interpreted in granulometry curves, shown in Figure 5. It can be seen that each compact has a different pore size distribution, which implies that the particle size used to fabricate the compacts directly influences the formation of pores, since they all followed low-temperature sintering conditions for each particle size used. The compact that presented a higher volume fraction with respect to the large pore size was the 75–106 µm compact, having pores with a size greater than 100 µm, while the 20–45 µm compact presented a higher number of pores with small sizes, from 15 µm to 40 µm. However, all the compacts showed the pattern of presenting the highest volume fraction with pore sizes from 20 µm to 60 µm. In a previous study, densification was linked to pore size as a function of the particle size used and attributed to sintering time and temperature [35].

In Table 2, all values of d_10_, d_50_ and d_90_ are given in addition to the span obtained for each compact, the d_50_ is the median pore size below which 50% of the fact that not all sample components have the same size. The d_90_ is the pore size below which 90% of the pores are contained and similarly d_10_.

It is observed that the values are very close to each other for all the compacts; the biggest difference is in d_90_ from 75 to 106 µm, since there is a difference of approximately 30 µm. This is because for these values, there is already a greater number of data contained in this calculation. When analyzing the span, it shows that the amplitude goes according to what was obtained in the accumulated fraction curves, the pore sizes obtained follow the trend of the particle size, this in order of all the particle sizes used. The difference is mostly reflected when going from the compact with a smaller particle size (20–45 µm) to the compact with a larger size (75–106 µm).

### 3.2. Permeability Analysis

The simulation determines the percentage of the total volume of the compact that is occupied by pores [36,37]. These pores will be filled with Ag or air if they are pores remaining after the infiltration process. If only the volume of the pores that are interconnected is considered, the software classifies it as effective porosity and the simulation flow runs through those spaces. The fluid velocity in the pore compact is proportional to the hydraulic gradient through the permeability coefficient, thus the software does not consider it as an intrinsic property and assigns velocity units to it. The flow lines are governed by a color system indicating the velocity at which the fluid passes through the pores; the red lines indicate where the fluid velocity is faster and the blue lines indicate where velocity is slowest.

**Figure 6:**
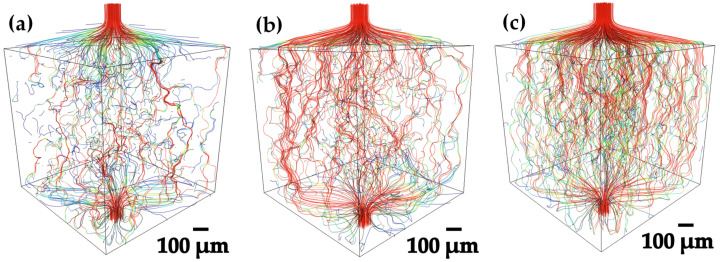
3D volume rendering of permeability simulations for the flow of Ag through the pores of Ti6Al4V compacts: (**a**) 20–45 µm, (**b**) 45–75 µm and (**c**) 75–106 µm.

Figure 6a–c shows the flow lines through the porous sample obtained from the permeability simulations for each compact worked at 1100 °C, which was the actual temperature at which the Ag infiltration was performed, showing the difference in viscosity behavior with respect to the interaction of the liquid/solid interface between Ag and Ti6Al4V. The 20–45 µm compact, that have a greater number of smaller pores (10 µm to 60 µm) with 25% of volume fraction. It is found that the liquid Ag infiltrates the compact slowly, since despite having a good level of interconnectivity, the reduced spaces of the channels make it more difficult for the liquid Ag to pass through, which can be seen by the fact that there are many blue flow lines and very few red ones. In the 45–75 µm compact, quite a drastic change is noticed, since in this compact there is a higher concentration of pores with sizes from 30 µm to 80 µm, reaching a volume fraction of 21%. However, in this compact, there is a small fraction of 0.003 of pores with size 100 µm, besides the interconnectivity of the pores occurred in a higher percentage and the tortuosity in the channels formed was reduced. The flow lines obtained are mostly red and green, which indicates a higher velocity and a reduction in the level of tortuosity, respectively. Nevertheless, there are still blue lines, which is interpreted as interconnected pores that hinder the passage of the liquid Ag, even when the infiltration temperature is increased and the viscosity is reduced. For the 75–106 µm compact, a concentration similar to the 20–45 µm compact was obtained, with pore sizes ranging from 20 µm to 70 µm. However, there is a concentration of large pores ranging from 80 µm to 170 µm, with a volume fraction of 0.044, which led to a direct improvement in the permeability levels for this compact, because having larger pores makes the flow of Ag easier, since the levels of permeability are greatly reduced. This led to a direct improvement in the permeability levels for this compact, because having larger pores makes the flow of Ag easier, since the levels of tortuosity generated are reduced by a large proportion. It was found that infiltration depends directly on the quantity and size of the pores generated. The interconnectivity plays an important role in infiltration as can be seen in permeability simulations, but it is worth noting that despite obtaining greater interconnectivity in the 20–45 µm compact in comparison with the 45–75 µm compact, better infiltration results were obtained, due to the aforementioned phenomenon. For the 75–106 µm compact, the pore size and interconnectivity results were very favorable.

**Figure 7:**
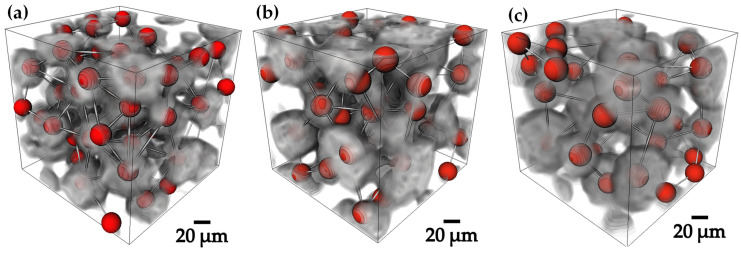
3D rendered skeletons showing nodes and segments of the sintered Ti6Al4V compacts: (**a**) 20–45 µm, (**b**) 45–75 µm and (**c**) 75–106 µm.

A 3D skeleton was made for all compacts with different particle size Figure 7a–c to ascertain whether the levels of interconnectivity were good [38], as this was required to perform the infiltration, in the generated skeleton images both lines and nodes can be observed, the lines correspond to the channels generated in the compact and are called “segments” and the “nodes” are the critical points where the lines converge and connect, generating different paths and interconnectivity between the channels [39]. Table 3 shows the values obtained for nodes and segments for each compact.

The 20–45 µm compact had the lowest number of nodes and segments; however, it can be seen that, due to the particle size, the channels are of a very small size, unlike the 45–75 µm compact where the main difference lies in the size of the channels. Although there are fewer, they are completely affected by the particle size, since they are larger, and this generates a better permeability in the compact. For the 75–106 µm compact, 21,889 nodes and 37,577 segments were obtained, being the compact with the highest number of formed and interconnected channels, in addition to having a good percentage of channels with a large size.

### 3.3. Dilatometry Analysis

**Figure 8:**
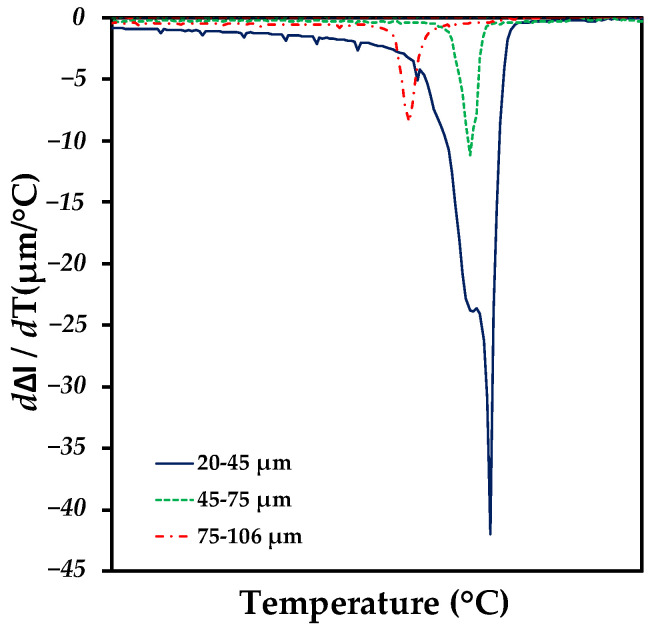
Infiltration curves of Ti6Al4V/xAg compacts showing exact temperature points where pores are filled with Ag.

The displacement was directly plotted as a function of temperature, allowing us to know the exact temperature point at which the Ag infiltrates in each compact and the range in which the Ag infiltrates completely in all the pores. Figure 8 shows the dilatometry curves for each compact where the infiltration range for all the compacts is affected between 950 °C and 970 °C. The melting of the Ag is reflected immediately upon reaching its theoretical melting point at 950 °C. However, the displacement is still affected as a function of the temperature increase, since the equipment takes the parameters of how the porosity of the compact is being filled by the Ag. All the compacts infiltrated at different temperature ranges; the compact that infiltrated first was the 75–106 µm compact, having its critical infiltration point at 953.6 °C (this is understood as the Ag occupied all the available empty spaces). Nonetheless, the displacement activity started from 950 °C—this completely governed as a function of the porosity and tortuosity generated.

Wettability is a factor that can be discarded, since, as mentioned by Assael M.J. et al., the viscosity of Ag does not change much as a function of the temperature range used, which was previously proven in permeability simulations [29]. The 45–75 µm compact infiltrated at 960.5 °C and its range of displacement activity started at 956.8 °C. On the other hand, the 20–45 µm compact was the one most affected by displacement, it was found that this one had two critical points of infiltration starting to infiltrate at 960.9 °C and finishing infiltration at 962. 9 °C, due to the fact that the amount of pores with a small size was seen in greater proportion in this compact once sintered. In addition to the tortuosity obtained with a large number of nodes generated but with channels of reduced size, making it difficult for the Ag to fill the pores in their entirety excluding the isolated pores.

### 3.4. Ag Infiltration

The way of working the new phases in the analysis of the microtomographies changes, since the contrasts of the initial image made it necessary to adjust the thresholds of the images and thus obtain a binary image where the remaining pores could be visualized, the threshold adjustment levels of the images depended on each compact worked, since each one had a different number of voxels [40]. However, the results in volume fraction directly agree with the data taken before performing the Ag infiltration, taking into account a 100% pore volume fraction since the beginning of the analysis.

**Figure 9:**
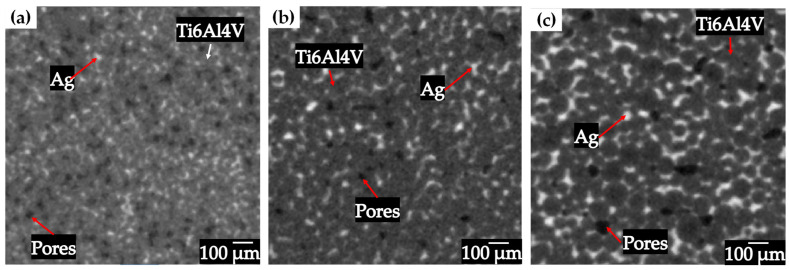
2D virtual slices of Ti6Al4V/xAg infiltrated: (**a**) 20–45 µm, (**b**) 45–75 µm and (**c**) 75–106 µm.

Ag infiltration was performed on Ti6Al4V compacts and 3D images of the infiltrated compacts were acquired by microtomography, as shown in Figure 9a–c. The difference in densities is reflected in the colors recorded in the images, the Ti6Al4V particles are recorded in dark gray, while the infiltrated Ag was recorded in light gray and the remaining pores are completely black. It is found that the infiltration process reached to fill most of the interconnected pores and leaving empty the remaining pores, since being completely isolated, they are not reached by the Ag and are completely empty. The image analysis process followed the same processing route that was used for the images of the pre-sintered compacts, the difference was in the Ag phase and how the images were processed with this new variable.

The compact with the highest volume fraction of Ag infiltrated is the 75–106 µm compact with 27.91% infiltrated. This due to the remaining porosity after sintering as discussed above in the 3D image analysis of the pre-sintered compacts. Numerical flow simulations and permeability analysis also agreed that this porous compact presented the best conditions for the infiltration process. The 20–45 µm and 45–75 µm compacts presented a lower volume fraction of Ag infiltrated, no more than 21% as shown in Table 1. However, they are still good infiltration levels to work with. All the compacts had a remaining porosity of less than 2% in volume, which indicates that the conditions worked in the pre-sintering process were optimal because they allowed to generate a good interconnectivity and tortuosity in the compacts [41,42].

**Figure 10:**
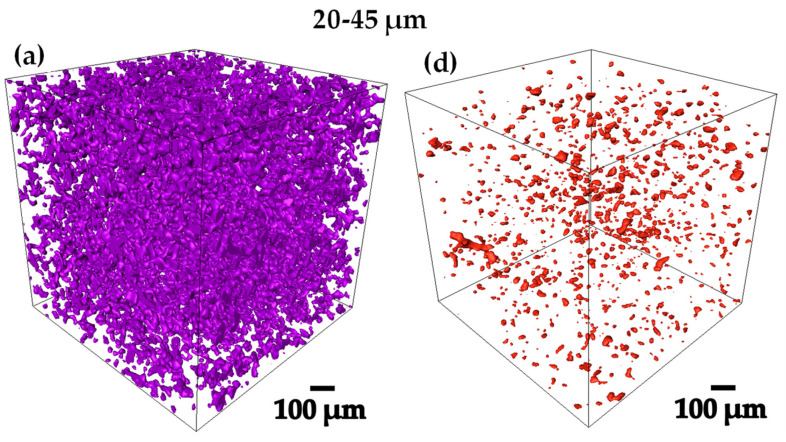

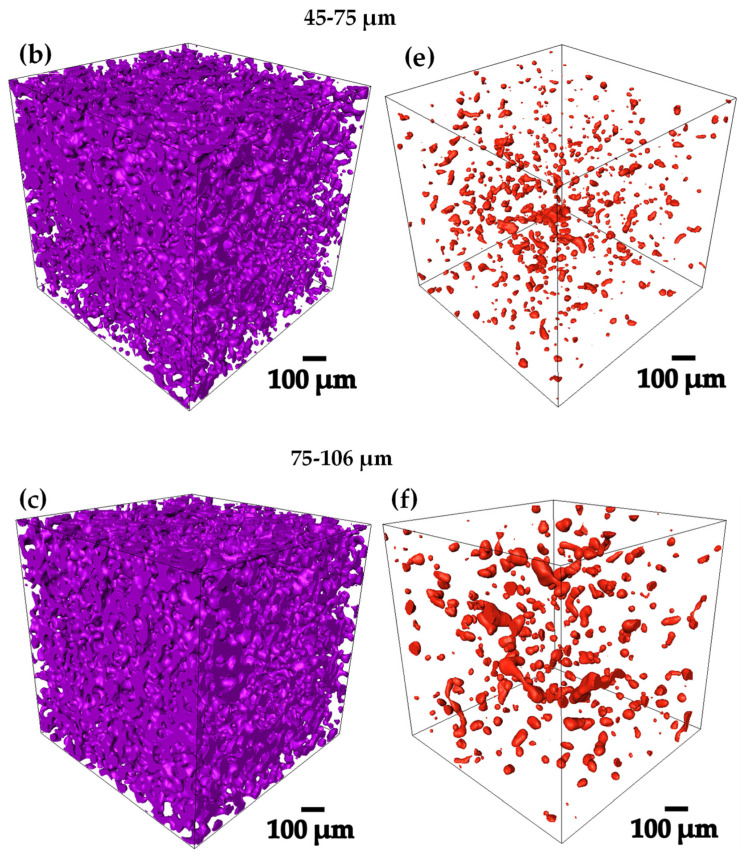
3D rendering of the infiltrated compacts showing 2 different phases (**a**–**c**) show the Ag distribution and (**d**–**f**) show the remaining pores distribution obtained.

In Figure 10a–f the 3D renderings of the Ag infiltrated in the compacts and the remaining porosity obtained after the process can be observed qualitatively. However, the results in volume fraction agrees directly with the data taken before performing the Ag infiltration, taking into account a 100% in volume from the beginning of the analysis.

**Figure 11:**
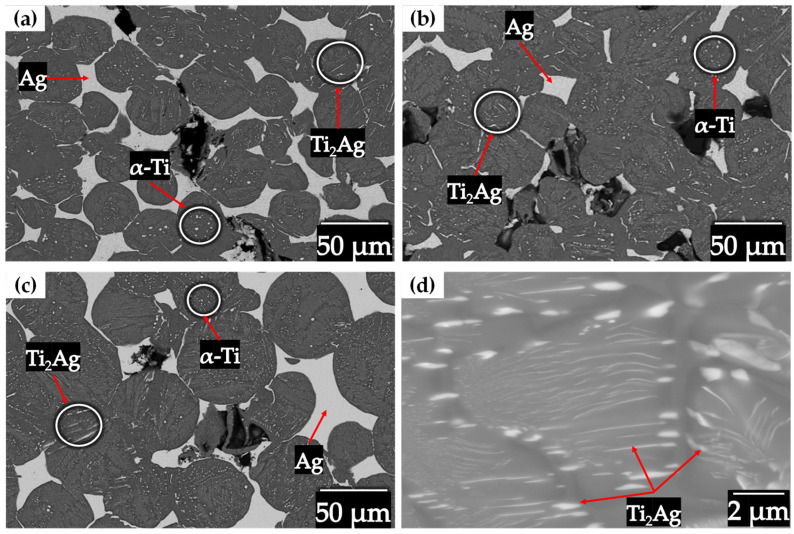
Backscattered electron image of the Ti6Al4V/xAg compacts: (**a**) 20–45 µm, (**b**) 45–75 µm, (**c**) 75–106 µm and (**d**) Ti_2_Ag lamellae inside of Ti6Al4V particles.

Figure 11 visualizes the microstructure in each compact after the infiltration process, for all compacts the same microstructure was obtained, composed by the phases: Ag, Ti_2_Ag and α-Ti. The Ti2Ag and α-Ti phases were found diffused within the titanium particles in the form of lamellae and small islands. The diffusion of Ag into the solid Ti6AlV particles generetes the formation of lamellae from houndreds of nanometers to a few microns of Ti_2_Ag phases, as shown in Figure 11d. The diffusion of some atoms into the cristalyne net of Ti6AlV phase induces the stabilization of α-Ti. This has been reported on Ti-Ag alloys fabricated by either powder matallurgy [14] or melting [43]. Shi et al., attributed that the antibacterial behavior is reflected by the Ti_2_Ag phase although the Ag phase also have antibacterial activity due to the release of ions, therefore all the compacts are considered antibacterial [44]. There are two points of view regarding the mechanisms of antibacterial action Ag. The first and widely accepted is the one related to the released metal ions killing bacteria, in where the dissolution of silver ions may also cause free radicals on the titanium surface that destroy the bacterial structure and so generate the silver ion sterilization [45,46]. The second is related to the contact sterilization; it is considered that the presence of Ag particles on the surface of the metal can generate normal physiological metabolic disorders of bacterial cell membrane, thus, leading to bacteria death [47]. Shi et al. [44] demonstrated that the precipitation of Ti_2_Ag in a contact sterilization mode plays a major role in the antibacterial ability of the Ti- Ag alloy than the silver ion release. The volume fraction of Ag obtained after the infiltration process are sufficient to have a good bactericidal rate since small amounts of Ag promote antibacterial activity [48].

### 3.5. Compression Tests

**Figure 12:**
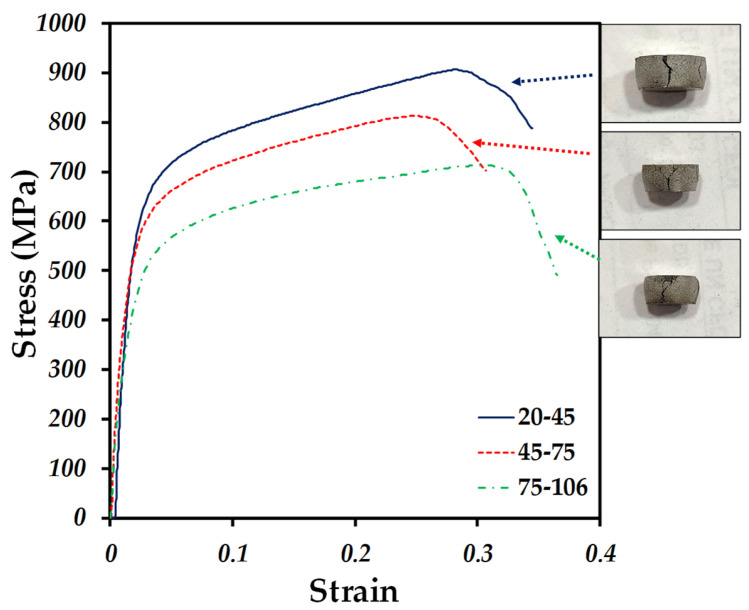
Stress–strain compression curves of Ti6Al4V/xAg with different particle size showing the fractures for each compact.

**Figure 13:**
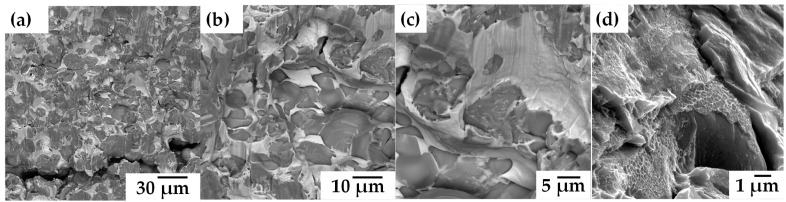
SEM images of fractured samples after compression tests at different magnifications; (**a**) 1000×, (**b**) 3000×, (**c**) 5000× and (**d**) 20,000×.

The infiltrated compacts were taken to the fracture point in order to know their mechanical resistance to compression. Figure 12 shows the compacts after the test and how the fracture propagated, which propagated through all the interparticle necks formed and also through the titanium particles. Figure 13a shows that the fracture propagation took place through the particles because a higher energy is required to break the necks formed, which was reflected in all the compacts [49]. It was also shown that Ag is mainly deformed during the compression tests, which brings a larger ductility to the composite (Figure 13b,c). In addition, it was observed that Ag covering the Ti6AlV particles also is break down when the strenght of the Ag reached their limit, Figure 13d. This suggests that the fracture behavior is composed of two mechanicsms, the first one is the plastic deformation of the Ag that covers the Ti6AlV particles that cans also be fractured. The second one leads to the fracture propagation once the limit of particles necks is reached, which turns in the fracture of composites.

In the strain-stress curves obtained from the compression tests, the compact with the best mechanical resistance to compression was the 20–45 µm compact, since having a greater number of particles forming necks generates a greater mechanical resistance.

Table 4 shows the yield stress (σ_y_) and Young’s modulus (E) for each compact. The 20–45 µm compact had a yield stress of 504 MPa and a Young’s modulus of 38 GPa, while the compact that had a lower mechanical resistance to compression was the 75–106 µm compact, due to the opposite case of the compact with smaller particle size, Since it has a smaller contact area between the particles, less interparticle necks are generated, which favors this behavior, resulting in a yield stress of 403 MPa and a Young’s modulus of 23 GPa. It is observed that the infiltration of Ag does not greatly influence the mechanical properties because the compacts have a behavior characteristic of compacts with those particle sizes.

The 75–106 µm compact, despite having a lower mechanical strength, is the option that would be the most viable for use in a biomedical prosthesis, since its mechanical properties are the most similar to those of human bones. Donald T. et al. reported values of Young’s modulus (E) for human bones in a range of 15 to 25 MPa, depending on the age, diet and physical routine of the patient [50]. Thus, the 75–106 µm compact falls within the range of mechanical properties of real bone; therefore, at the time of anchoring between the human bone and the compact of these characteristics there will be no mechanical decompensation, thus ensuring the life of the compact and the longevity of the user’s bone, avoiding compromising both parts [51].

## 4. Conclusions

The pressureless gravity infiltration of Ag into porous Ti6Al4V solid compacts with different particle sizes sintered at low-temperature has been successfully performed. It has been demonstrated that the larger the pore size, the better the filling in the gravity infiltration process, as the higher the liquid velocity, the better the permeability of the compact. In addition, the diffusion of Ag in the Ti6Al4V particles leads to the formation of the Ti2Ag phase in the form of lamellae.

The 3D analysis by microtomography made it possible to determine the pores’ features through which the liquid silver passes during the infiltration process. The best infiltration results were obtained in the compacts with a particle size of 75–106 µm, since they allowed a higher densification and a faster filling of the compact, which benefited the mechanical properties of this compact to be the most similar to those of human bones.

## Figures and Tables

**Table 1:** Volume fraction of different phases of the porous compacts and composites after infiltration process.

Sample	Vol. fraction of Ti6AlV (%)	Vol. Fraction of Pores (%)	Vol. Fraction of Ag (%)	Fraction of Pores Filled with Ag (%)
Before infiltration				
20–45 μm	82.59	17.41	--	--
45–75 μm	78.71	21.29	--	--
75–106 μm	70.38	29.62	--	--
After infiltration				
20–45 μm	82.59	1.69	15.60	89.6
45–75 μm	78.71	1.24	20.04	94.13
75–106 μm	70.38	1.70	27.91	94.23

**Table 2:** Cumulative values of sintered compacts of Ti6Al4V.

Particle Size (μm)	d_10_ (μm)	d_50_ (μm)	d_90_ (μm)	Span
20–45	9.4920	22.6984	44.5297	1.5436
45–75	16.4927	36.6467	56.7814	1.0993
75–106	13.5212	33.8838	87.8704	2.1942

**Table 3:** Segments and nodes generated in sintered Ti6Al4V compacts.

Particle Size (µm)	Segments	Nodes
20–45	16,306	11,643
45–75	18,706	21,565
75–106	21,889	37,577

**Table 4:** Modulus of elasticity and yield stress obtained in compression tests of Ti6Al4V/xAg compacts.

Particle Size (μm)	Yield Stress σ_y_ (MPa)	Young’s Modulus E (GPa)
20–45	504	38
45–75	463	30
75–106	403	23

## Data Availability

The data presented in this study are available on request from the corresponding author. The data are not publicly available due to the fact that are part of an ongoing study.
